# Glucose Increases Hepatic Mitochondrial Antioxidant Enzyme Activities in Insulin Resistant Rats Following Chronic Angiotensin Receptor Blockade

**DOI:** 10.3390/ijms231810897

**Published:** 2022-09-17

**Authors:** Jose A. Godoy-Lugo, Max A. Thorwald, Dora A. Mendez, Ruben Rodriguez, Daisuke Nakano, Akira Nishiyama, Rudy M. Ortiz

**Affiliations:** 1 Department of Molecular & Cell Biology, School of Natural Sciences, University of California, Merced, CA 95343, USA; 2 Leonard Davis School of Gerontology, University of Southern California, Los Angeles, CA 90089, USA; 3 Department of Pharmacology, Kagawa Medical University, Kagawa 761-0793, Japan

**Keywords:** 4-HNE, AT1, antioxidants, collagen, fibrosis, NAFLD

## Abstract

Non-alcoholic fatty liver disease (NAFLD) affects up to 20% of the world’s population. Overactivation of the angiotensin receptor type 1 (AT1) contributes to metabolic dysfunction and increased oxidant production, which are associated with NAFLD and impaired hepatic lipid metabolism. Nuclear factor erythroid-2-related factor 2 (Nrf2) regulates the expression of antioxidant phase II genes by binding to the antioxidant response element (ARE); however, the mechanisms by which AT1 contributes to this pathway during the progression of NAFLD remain unresolved. To investigate hepatic Nrf2 response to a hyperglycemic challenge, we studied three groups of rats (male, 10-weeks-old): **(1)** untreated, lean Long Evans Tokushima Otsuka (LETO), **(2)** untreated, obese Otsuka Long Evans Tokushima Fatty (OLETF), and **(3)** OLETF + angiotensin receptor blocker (OLETF + ARB; 10 mg olmesartan/kg/d × 6 weeks). Livers were collected after overnight fasting (T0; baseline), and 1 h and 2 h post-oral glucose load. At baseline, chronic AT1 blockade increased nuclear Nrf2 content, reduced expression of glutamate-cysteine ligase catalytic (GCLC) subunit, glutathione peroxidase 1 (GPx1), and superoxide dismutase 2 (SOD2), mitochondrial catalase activity, and hepatic 4-hydroxy-2-nonenal (4-HNE) content. The expression of hepatic interleukin-1 beta (IL-1β) and collagen type IV, which are associated with liver fibrosis, were decreased with AT1 blockade. Glucose increased Nrf2 translocation in OLETF but was reduced in ARB, suggesting that glucose induces the need for antioxidant defense that is ameliorated with ARB. These results suggest that overactivation of AT1 promotes oxidant damage by suppressing Nrf2 and contributing to hepatic fibrosis associated with NAFLD development.

## 1. Introduction

Non-alcoholic fatty liver disease (NAFLD) is the most common chronic hepatic disease worldwide [1]. NAFLD is a spectrum of liver injury, ranging from simple steatosis and non-alcoholic steatohepatitis (NASH) to fibrosis and cirrhosis [2]. Obesity and metabolic dysfunction (i.e., impaired lipid metabolism) are principal contributing factors for the development of NAFLD [3]. A commonality among these is the potential overactivation of the renin-angiotensin system (RAS), which stimulates its effects through the angiotensin II (Ang II) receptor type 1 (AT1) [4,5]. An updated framework for the pathogenesis of NAFLD and NASH suggests that increased availability of metabolic substrates (i.e., glucose and fatty acids) surpasses their processing rate, leading to their accumulation, and consequentially promoting the formation of oxidants [6]. Oxidants are also largely produced by mitochondria, generating hydrogen peroxide (H_2_O_2_) and peroxynitrite (ONOO−). Increased cellular oxidants promote endoplasmic reticulum (ER) stress, mitochondrial dysfunction, and inflammasome activation, which are shown to worsen NAFLD [7,8,9].

Glutathione (GSH) is a tripeptide that is present in all mammalian cells [10] and primarily responsible for the reduction of harmful oxidant species and conjugation to xenobiotics [11]. Low GSH levels are associated with metabolic disease, including type 2 diabetes (T2D) [12] and NAFLD [13]. Conversely, oral GSH supplementation decreased circulating markers of liver and oxidative damage [14] by participating in the reduction of hydroperoxides [15]; accordingly, dietary GSH consumption is also suggested to reduce body mass in patients with metabolic syndrome [16]. The rate limiting step in GSH production is the γ-glutamylcysteine substrate, generated by glutamate-cysteine ligase (GCL), which is comprised of two subunits, catalytic (GCLC) and modifier (GCLM) [17]. GCLC catalyzes the production of GSH [18] while GCLM is not enzymatically active, but increases the activity of GCLC by increasing the affinity to its substrate [19]. Oxidized GSH (GSSG) is converted back to GSH by glutathione reductase (GR) to replenish the GSH pool [17]. GSH is also found in plasma, where it can be broken down by other tissues, through γ-glutamyl transpeptidase (GGT) for uptake of cysteine for GSH production [20]. Most of the circulating GSH is secreted by the liver [21], therefore hepatic impairments of GSH production and recycling are suggested to contribute more prominently to NAFLD pathophysiology [17].

Nuclear factor erythroid-2-related factor 2 (Nrf2) is crucial for the maintenance of cellular redox homeostasis [22]. Nrf2 regulates the expression of genes responsible for the production of GSH, xenobiotic detoxification, neutralization of oxidants, and NADPH replenishment [23]. Nrf2 signaling is required to support mitochondrial function [22] and for the detoxification of oxidants in the cytosol and ER [24]. Nrf2 regulates the antioxidant enzymes catalase (CAT), glutathione peroxidase (GPx), and superoxide dismutase (SOD), which counteract oxidant production [25]. Loss of Nrf2 activity also caused lipid accumulation in the liver [26], while Nrf2 overexpression decreased hepatic lipid levels [27], suggesting a beneficial role for Nrf2 in the fatty liver.

During diabetes, nuclear translocation of Nrf2 is reportedly impaired, preventing the induction of its target genes and resulting in tissue-specific injury [28]. We have shown that the early onset of hyperglycemia, characteristic of OLETF rats, is associated with reduced Nrf2 activity in the heart [29]. Moreover, an acute glucose challenge increased Nrf2 levels, while chronic blockade of AT1 decreased them, suggesting that the need to induce antioxidant defenses in response to acute hyperglycemia is reduced. However, the behavior of hepatic Nrf2 in response to a glucose challenge, and the contributions of AT1, have not been examined. Therefore, the present study of Nrf2 during development of NAFLD and overactive RAS, will provide insights into Nrf2 inducibility, AT1 activation, and their relationship with NAFLD. We investigated the levels of Nrf2, genes of GSH regulation, and mitochondrial antioxidant proteins during hepatic steatosis, including chronic AT1 blockade. Furthermore, the dynamic effects of nutrient overload (through an acute glucose challenge) on Nrf2 inducibility during NAFLD are scarce. Therefore, the hyperglycemic challenge performed in this study enhances the understanding of potential injury mechanisms induced by high glucose via altered antioxidant responses that may promote NAFLD.

## 2. Results

To better recognize the differences between the chronic, static effects associated with chronic AT1 blockade following an overnight fast (captured by differences at T0 or baseline) and the acute, dynamic responses to an acute glucose challenge (captured by the changes over the measurement time points), the results were separated into two sections designated by **(I)** static effects and **(II)** dynamic responses, respectively.

### 2.1. Chronic, Static Effects of ARB Treatment

#### 2.1.1. OLETF Rats Present with Hepatic Hyperglycemia and Hypertriglyceridemia

To confirm the NAFLD phenotype in the OLETF rat, liver glucose and triacylglycerol (TAG) levels were compared across the groups. Mean hepatic glucose (mg/dL/μg protein) content was 137% greater in OLETF (2.31 ± 0.43) than LETO (0.97 ± 0.24) and 58% lesser in OLETF + ARB (1.54 ± 0.13) than OLETF. Similarly, mean hepatic TAG (mg/μg protein) content was 125% greater in OLETF (10.16 ± 1.83) than LETO (4.52 ± 0.47) and 20% lesser in OLETF + ARB (8.10 ± 1.07).

#### 2.1.2. AT1 Blockade Increases Nuclear Hepatic Nrf2 Levels 

**Figure 1:**
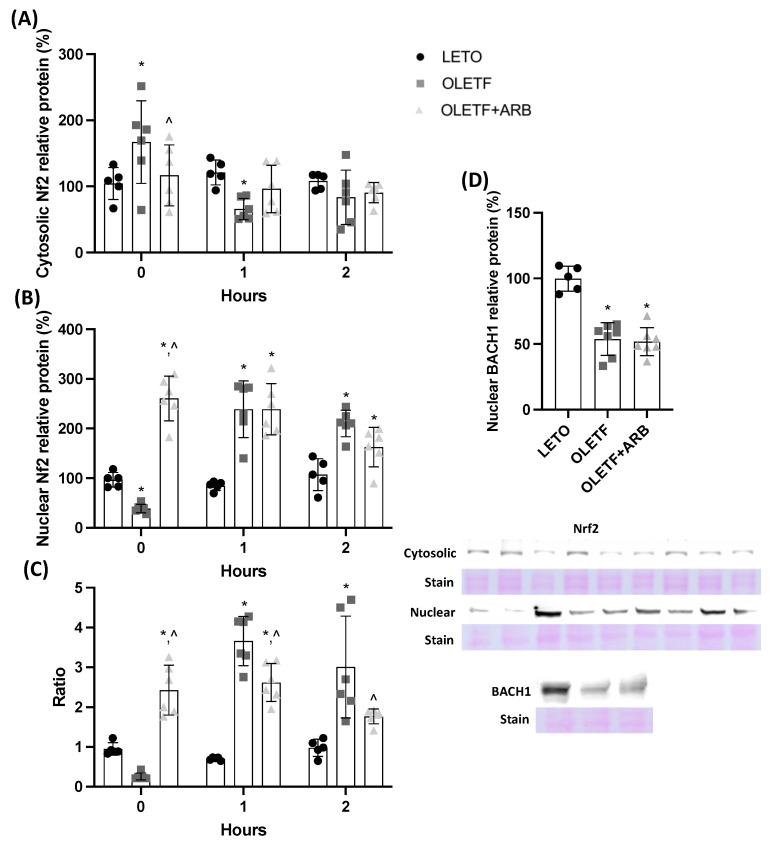
**Nuclear Nrf2 content is increased with AT1 blockade.** Mean ± SD hepatic nuclear factor erythroid-2-related factor (Nrf2) protein levels in (**A**) cytosol, (**B**) nucleus, (**C**) their calculated nuclear:cytosolic ratio, and (**D**) BACH1 nuclear protein levels before and during the glucose load in Long Evans Tokushima Otsuka (LETO), Otsuka Long Evans Tokushima Fatty (OLETF), and OLETF + ARB (ARB) rats. * significantly different *from LETO (p < 0.05); ^ significantly different from OLETF (p < 0.05)*.

Studies suggest that Nrf2 signaling can be impaired during metabolic alterations [28,30]. We measured cytosolic and nuclear protein levels of Nrf2 and calculated the ratio of nuclear:cytosolic to suggest changes in its translocation. Cytosolic Nrf2 levels were 61% greater in OLETF than LETO and levels in OLETF + ARB were 45% lesser than OLETF (Figure 1A). Nuclear Nrf2 levels were 59% lesser in OLETF than LETO, while levels in OLETF + ARB were 63% and 85% greater than LETO and OLETF, respectively (Figure 1B). The nuclear:cytosolic ratio of Nrf2 in OLETF + ARB was 154% and 860% greater than LETO and OLETF, respectively (Figure 1C). Finally, the nuclear Nrf2 suppressor BACH1 was 48% lesser in both OLETF and OLETF + ARB compared to LETO (Figure 1D).

#### 2.1.3. Hepatic Expressions of GCLC and GR Were Decreased with AT1 Blockade 

**Figure 2:**
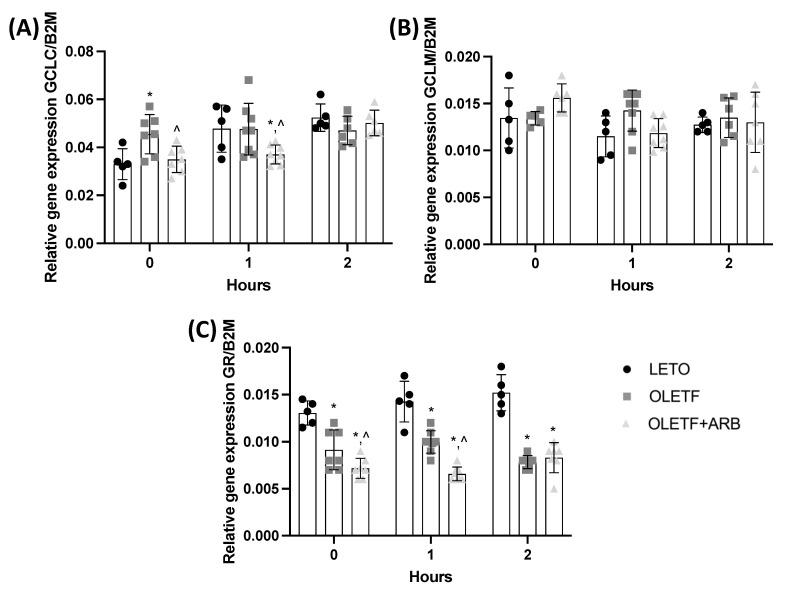
**Expression of glutathione genes is reduced with AT1 blockade.** Mean ± SD mRNA levels of hepatic (**A**) glutamate-cysteine ligase catalytic (GCLC), (**B**) glutamate-cysteine ligase modifier (GCLM), and (**C**) glutathione reductase (GR) before and during the glucose load in Long Evans Tokushima Otsuka (LETO), Otsuka Long Evans Tokushima Fatty (OLETF), and OLETF + ARB (ARB) rats. * significantly different *from LETO (p < 0.05); ^ significantly different from OLETF (p < 0.05)*.

The liver is a unique organ because it produces cysteine, a GSH precursor [31]. Measuring the gene expression of Nrf2-target enzymes that regulate GSH synthesis and cycling can provide insight into GSH homeostasis during NAFLD and after chronic AT1 blockade. Basal GCLC expression in OLETF was 38% greater than LETO and was 23% lesser in OLETF + ARB than OLETF (Figure 2A). No differences in CGLM expression were detected among the groups (Figure 2B). GR expression in OLETF and OLETF + ARB was 32% and 46% lesser than LETO, respectively, while OLETF + ARB was 22% lesser than OLETF (Figure 2C). 

#### 2.1.4. Total Hepatic 4-Hydroxynonenal Levels and Mitochondrial Catalase Activity Were Decreased with AT1 Blockade

**Figure 3:**
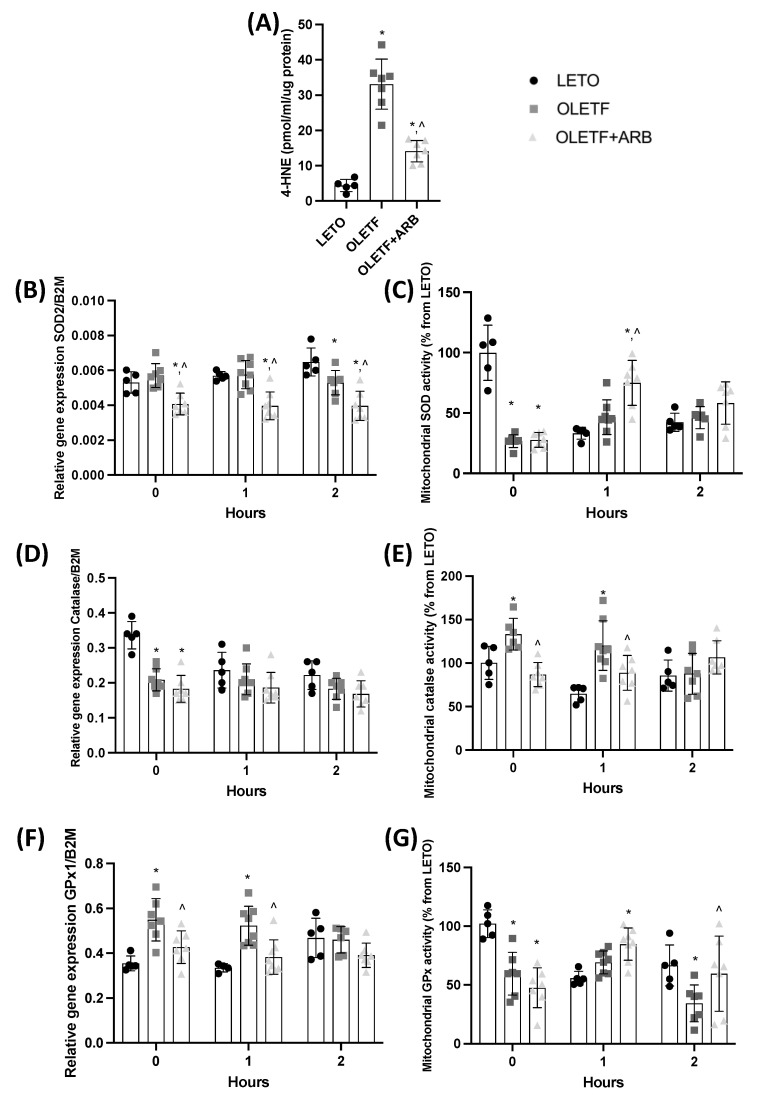
**Lipid peroxidation is decreased after AT1 blockade.** Mean ± SD hepatic (**A**) total 4-hydroxy-2-nonenal (4-HNE) levels and (**B**) mRNA levels of superoxide dismutase 2 (SOD2), (**C**) activity levels of mitochondrial SOD, (**D**) mRNA levels of catalase, (**E**) activity levels of mitochondrial catalase, (**F**) mRNA levels of glutathione peroxidase 1 (GPx1), and (**G**) activity levels of mitochondrial GPx before and during the glucose load in Long Evans Tokushima Otsuka (LETO), Otsuka Long Evans Tokushima Fatty (OLETF), and OLETF + ARB (ARB) rats. * significantly different *from LETO (p < 0.05); ^ significantly different from OLETF (p < 0.05)*.

Increased oxidant levels are reduced by the activation of antioxidant enzymes (e.g., catalase, GPx, and SOD) [32]. Hepatic 4-hydroxy-2-nonenal (4-HNE) is detected during NAFLD and serves as a biomarker of increased peroxidation injury [33]. Since the basal expression of the GSH regulatory genes was reduced, we wanted to assess the levels of 4-HNE and the response of the mitochondria to potentially increased oxidation. Total 4-HNE levels in OLETF and OLETF + ARB were 650% and 220% greater, respectively, than LETO, while OLETF + ARB levels were 57% lesser than OLETF (Figure 3A). SOD2 expression in OLETF + ARB was 25% and 30% lesser than LETO and OLETF, respectively (Figure 3B). Mitochondrial SOD activity levels in OLETF and OLETF + ARB were 73% and 72% lesser, respectively, than LETO (Figure 3C). Catalase expression in OLETF and OLETF + ARB was 38% and 46% lesser, respectively, than LETO (Figure 3D). Mitochondrial catalase activity levels in OLETF were 36% greater than LETO, and 33% lesser in OLETF + ARB compared to OLETF (Figure 3E). GPx1 expression in OLETF was 57% greater than LETO, while expression in OLETF + ARB was 31% lesser than OLETF (Figure 3F). Mitochondrial GPx activity levels in OLETF and OLETF + ARB were 42% and 54% lesser, respectively, than LETO (Figure 3G). 

#### 2.1.5. Hepatic Expression of IL-1β and Plasma Levels of Col4 Decreased with AT1 Blockade 

**Figure 4:**
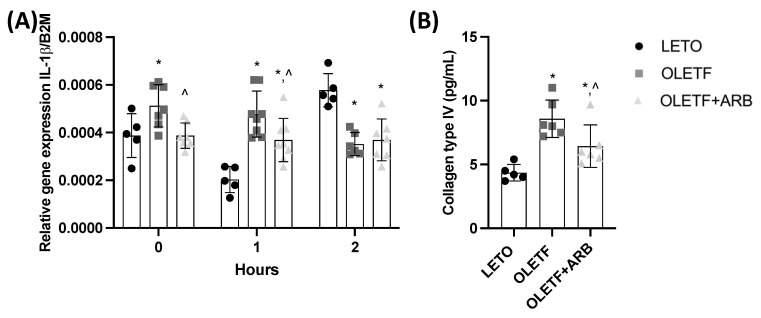
**Fibrosis markers are decreased with AT1 blockade.** Mean ± SD hepatic (**A**) mRNA levels of interleukin-1 beta (IL-1β) and (**B**) collagen type IV (COL-4) before and during the glucose load in Long Evans Tokushima Otsuka (LETO), Otsuka Long Evans Tokushima Fatty (OLETF), and OLETF + ARB (ARB) rats. * significantly different *from LETO (p < 0.05); ^ significantly different from OLETF (p < 0.05)*.

Interleukin-1 beta (IL-1β) contributes to hepatic injury and fibrosis [34] and AT1 blockade has been shown to decrease hepatic fibrosis in patients with NAFLD [35]. Given that our study captures the early onset of NAFLD, we measured the expression of IL-1β and the plasma biomarker, collagen IV (Col4), to assess the effects of AT1 blockade on liver health. Hepatic IL-1β expression in OLETF was 32% greater than LETO, while the expression in OLETF + ARB was 29% lesser than OLETF (Figure 4A). Plasma Col4 levels in OLETF and OLETF + ARB were 107% and 49% greater, respectively, than LETO, but 28% lesser in OLETF + ARB compared to OLETF (Figure 4B).

### 2.2. Acute, Dynamic Responses to Glucose Challenge

#### 2.2.1. Glucose Stimulated Nrf2 Translocation in OLETF 

At T1, cytosolic Nrf2 levels were 45% lesser in OLETF than LETO (Figure 1A), while nuclear Nrf2 levels in OLETF and OLETF + ARB were 181% greater than LETO (Figure 1B). The nuclear:cytosolic ratio of Nrf2 in OLETF and OLETF + ARB was 420% and 270% greater, respectively, than LETO (Figure 1C). At T2, nuclear Nrf2 levels were 96% and 51% greater in OLETF and OLETF + ARB, respectively, than LETO (Figure 1B). The ratio of Nrf2 in OLETF was 210% greater than LETO, while OLETF + ARB was 70% lesser than OLETF (Figure 1C).

#### 2.2.2. Hepatic GCLC, GCLM, and GR Expressions Remained Constant during the Acute Glucose Challenge 

In response to the glucose challenge, hepatic GR expression remained decreased in the OLETF and treatment groups, GCLM was unchanged, while GCLC was decreased with AT1 blockade. At T1, GCLC expression in OLETF + ARB was 22% lesser than OLETF (Figure 2A). No changes in GCLM in response to glucose were detected (Figure 2B). GR expression in OLETF and OLETF + ARB were 30% and 54% lesser, respectively, than LETO, and 34% lesser in OLETF + ARB than OLETF (Figure 2C). At T2, GR expression in OLETF and OLETF + ARB were 48% and 45% lesser, respectively, than LETO (Figure 2C). Additionally, GR expression in LETO was positively correlated (r = 0.997, *p* = 0.047) overtime during the glucose load.

#### 2.2.3. SOD and GPx Activities Increased in Response to the Glucose Challenge 

At T1, SOD2 expression in OLETF + ARB was 30% and 32% lesser than LETO and OLETF, respectively (Figure 3B), while mitochondrial SOD activity in OLETF + ARB was 127% and 63% greater than LETO and OLETF, respectively (Figure 3C). There were no detectable changes in catalase expression among the groups in response to the glucose challenge (Figure 3D). At T1, mitochondrial catalase activity in OLETF was 85% greater than LETO, and 26% lesser in OLETF + ARB than OLETF (Figure 3E). At T1, GPx1 expression in OLETF was 58% greater than LETO, while OLETF + ARB was 27% lesser than OLETF (Figure 3F). Mitochondrial GPx activity in OLETF + ARB was 53% greater than LETO, while at T2, GPx activity in OLETF was 49% lesser than LETO and was 42% greater in OLETF + ARB compared to OLETF (Figure 3G).

#### 2.2.4. Chronic ARB Treatment Stabilized the Expression of IL-1β in Response to a Glucose Challenge 

IL-1β expression in OLETF + ARB did not increase in response to glucose (Figure 4A). At T1, IL-1β expression in OLETF and OLETF + ARB was 135% and 80% greater, respectively, than LETO, while OLETF + ARB was 23% lesser than OLETF (Figure 4A). At T2, IL-1β expression in OLETF and OLETF + ARB was 39% and 35% lesser, respectively, than LETO (Figure 4A).

## 3. Discussion

The liver injury associated with NAFLD can present before MetS and T2D, all of which are associated with overactivation of RAS and oxidative stress [5,6,36,37]. Increased oxidant levels and lipid peroxidation contribute to the multifactorial pathogenesis of NAFLD [36]. Conversely, increases in antioxidant enzymes, SOD and GPx, can ameliorate oxidative injury and subsequently protect the liver [38,39]. In the present study, the mitochondrial activities of SOD and GPx were increased in the liver following a glucose load. After the onset of NAFLD, Nrf2 activity may be decreased, resulting in reduced antioxidant levels [6]. We found that chronic blockade of AT1 increased hepatic nuclear Nrf2 protein, which corresponded with decreased lipid peroxidation (4HNE), suggesting that overactivation of AT1 inhibits Nrf2 activity and subsequently promotes liver injury.

In addition to promoting antioxidants, Nrf2 can inhibit hepatic lipogenesis [40]. In our study, basal nuclear Nrf2 levels were decreased in non-treated OLETF rats and increased in response to the glucose load. This suggests that Nrf2 is chronically suppressed in our model of early NAFLD development, reducing antioxidant potential and leading to oxidative injury and NAFLD. The increase in Nrf2 translocation ratio in OLETF in response to an acute glucose challenge suggests that, at this stage of hepatic disease: **(1)** the liver can respond to nutrient overloads to compensate for potential insults derived from chronic hyperglycemia, and **(2)** acutely increased glucose induces an oxidizing environment that stimulates an Nrf2-mediated antioxidant response. Conversely, chronic blockade of AT1 statically increased nuclear Nrf2 and reduced hepatic 4-HNE, suggesting that the maintenance of elevated nuclear Nrf2 levels reduced oxidative injury. In the heart of older diabetic rats treated with ARB, Nrf2 was basally increased in the presence of reduced oxidative injury, while its target genes remained unchanged [41]. This comparison demonstrates that other factors may be suppressing Nrf2 activation. The basal decrease in BACH1 suggests that, in this particular case, the mechanism preventing Nrf2 ability to bind to DNA [42] is not BACH1-dependendant. This provides insight on the regulation of nuclear Nrf2 during the progression of NAFLD. Collectively, these data suggest that Nrf2 translocation is still inducible, but the downstream upregulation of antioxidant genes is potentially modulated by other factors, independent of BACH1.

Superoxide dismutase (SOD) catalyzes the dismutation of superoxide to hydrogen peroxide [43] and its dysregulation can cause cellular damage [44]. GPx1 uses GSH to catalyze the conversion of hydrogen peroxide to water [15]. Catalase also reduces hydrogen peroxide to water and oxygen. Elevated oxidants in the liver can increase hepatic mitochondrial catalase activity [45]. In this study, the GPx1 expression and mitochondrial catalase activity decreased with AT1 blockade [46], suggesting that overactivation of AT1 reduces antioxidants. This is supported by previous evidence showing that chronic blockade of AT1 protected against oxidative damage despite reduced catalase activity [47]. While low levels of antioxidant enzymes found in this study (i.e., catalase, GPx, and SOD) suggest that protection against oxidative stress is reduced, the reduction in lipid peroxidation indicates that the need for enhanced antioxidant defense was reduced. This is further substantiated by the reduced levels of Col4 and IL-1β as indicators of injury and inflammation. 

The decreased expressions of GCLC, GR, and GPx1 1 h post-glucose load in OLETF rats treated with ARB may reflect an environment with reduced levels of oxidants. Conversely, low GSH levels have been associated with NAFLD in humans [13], suggesting that the factors promoting steatosis (i.e., elevated Ang II, impaired lipid metabolism, impaired hepatic glycolysis, etc.) chronically may also compromise the liver. However, the progression of NAFLD in OLETFs at this age may not be sufficiently severe [48] to elicit profound impairment in antioxidant defenses. Nonetheless, while still in early stages of NAFLD [49], increased oxidant production may be sufficient to increase the demand for GSH [1]; however, as the condition progresses, the liver may lose the capacity to restore redox balance and respond to frequent insults, such as nutrient overload. Moreover, during the late-stages of T2D, the antioxidant response is reduced [50]. This suggests that during advanced NAFLD, GSH levels may be decreased due to its increased metabolism to reduce oxidants and not due to decreased cellular demand and/or synthesis [13]. 

The increased expressions of GCLC and GPx1 in OLETF suggest that more GSH production and oxidant clearance is needed during the onset of NAFLD [51] to counteract the increasing oxidant production [52]. However, the basal expression of GR was reduced in OLETF groups and remained so throughout the post-glucose load period. This suggests that GR expression may be decreased at early stages of NAFLD in MetS, reducing glutathione cycling, promoting an increased oxidative state [52]. Additionally, we observed a linear increase in GR after the glucose load only in the LETO, suggesting that GR upregulation is impaired in OLETF rats. This impaired response to glucose may promote glucotoxicity [53], further impairing the redox homeostasis. Thus, chronic AT1 blockade may not be sufficient to reverse the glucose-induced suppression of GR expression, yet it may promote antioxidation mechanisms through other pathways (e.g., SOD, catalase, reducing lipid availability) [49]. This also suggests that other mechanisms, such as pro-inflammatory pathways, likely contribute to MetS-associated liver injury and NAFLD progression independently of AT1 activation.

Oxidative injury can promote the progression of hepatic fibrosis [54]. While the contribution of inflammation to NAFLD is established, the impact of specific inflammatory cytokines is unresolved [55]. Elevated IL-1β is strongly associated with increased risk of NAFLD and liver fibrosis [34,55]. Our results demonstrated that chronic AT1 blockade decreased the basal expression of hepatic IL-1β, along with reducing TAG and 4-HNE levels. Additionally, the plasma marker Col4, which accurately represents the severity of hepatic fibrosis during NAFLD [56,57], was decreased with chronic AT1 blockade. This reduction may also represent amelioration of a pro-oxidative state produced by overactivation of AT1 [58,59]. In this study, plasma Col4 was decreased after chronic AT1 blockade, suggesting that the progression of liver fibrosis may be reduced or delayed [57]. The reduction in plasma Col4 with ARB treatment may also represent amelioration of a pro-oxidative state produced by overactivation of AT1 [58,59]. Accordingly, in older OLETF rats [60], AT1 blockade also decreased Col4, suggesting that, even during more advanced stages of NAFLD [48], chronic AT1 blockade improves the redox state in the liver and ameliorates liver fibrosis and potentially its progression.

In summary, these data suggest that overactivation of AT1, associated with hyperglycemia and hypertriglyceridemia, promotes hepatic oxidative injury by decreasing chronic nuclear levels of Nrf2. The reduction of oxidants, as indicated by the decreased 4-HNE levels, is likely achieved by increased mitochondrial catalase activity and GPx1 expression. These data suggest that in the liver of insulin-resistant rats with early stages of NAFLD, AT1 blockade increases nuclear Nrf2, potentially promoting antioxidant defenses. Furthermore, the benefits associated with chronic AT1 blockade translated to a more balanced response to the glucose excursions induced by the acute challenge. Our results support the idea that improved methods of early detection and intervention [61], in this case through AT1 blockade, may prevent the progression and severity of NAFLD derived from increased oxidant production [36,62]. 

## 4. Methods

### 4.1. Animals

All experimental procedures were reviewed and approved by the institutional animal care and use committees of Kagawa Medical University (Japan) and the University of California, Merced (USA). The OLETF rat is characterized by elevated Ang II and upregulated AT1, indicative of an overactivated RAS, making them a suitable model for the study of RAS-related metabolic alterations [63]. In this study, male, age matched, 10-week-old, lean strain-control Long Evans Tokushima Otsuka (LETO; 279 ± 7 g) and obese Otsuka Long Evans Tokushima Fatty (OLETF; 359 ± 3 g) rats (Japan SLC Inc., Hamamatsu, Japan) were used. The degree of NAFLD progression in OLETF can be estimated according to their age [48], hence, we started the study with rats at 10 weeks of age and finished at 16 weeks of age, which allowed for the examination of our targets during the development of fatty liver. Rats were assigned to the following groups (n = 5–8 animals/group/time point): **(1)** untreated LETO (n = 15), **(2)** untreated OLETF (n = 23), and **(3)** OLETF + angiotensin receptor blocker (OLETF + ARB; 10 mg olmesartan/kg/d × 6 weeks; n = 22). ARB (Daiichi-Sankyo, Tokyo, Japan) was administered by oral gavage suspended in carboxymethyl cellulose (CMC) to conscious rats and untreated rats were gavaged with CMC only [41]. Animals were maintained in groups of two to three animals per cage, given access to water and standard laboratory chow (MF; Oriental Yeast Corp., Tokyo, Japan), and maintained under controlled temperatures (23–24 °C) and humidity (~55%) with a light–dark cycle of 12–12 h. 

### 4.2. Glucose Challenge

To investigate the dynamic responses to a nutrient overload, animals were fasted overnight (12 h) prior to administration of a glucose challenge. Following the overnight fast, the animals representing baseline time point (T0) were sacrificed accordingly and the remaining animals were assigned to the designated timepoints: 1 h (T1) and 2 h (T2) after the oral glucose load (2 g glucose/kg). Staggered times were used to ascertain the correct timing for the following timepoint dissections. Vials containing EDTA and proteinase inhibitor cocktail (Sigma-Aldrich, St. Louis, MO, USA) were used to collect trunk blood. Livers were perfused, frozen in liquid nitrogen, and kept at −80 °C until analyzed. 

The T0 samples provide an indication of the chronic, static condition associated with the development of NAFLD following an overnight fast, while the inclusion of the time point measurements following an acute glucose bolus represent the acute, dynamic, molecular responses to a nutritional perturbation to assess the cellular behavior.

### 4.3. Plasma Marker of Hepatic Fibrosis

Plasma Col4 was measured as a biomarker of hepatic fibrosis using a commercially available kit (CIV ELISA, MyBioSource, San Diego, CA, USA, MBS732756), following the manufacturer’s instructions. All samples were analyzed in duplicate and only values that fell within percent coefficients of variability of less than 10% for all measurements were accepted.

### 4.4. Western Blots

A piece of frozen liver (40 mg) was homogenized in 100 μL of sucrose buffer for a multi-step extraction of cytosolic, mitochondrial, and nuclear fractions as previously described [28,64]. Briefly, a liver sample was homogenized in STM buffer, spun at 800 g for 15 min, and the pellet was saved. The recovered supernatant was further centrifuged at 11,000 g for 10 min to remove cellular debris, and re-centrifuged at 12,000 g for 5 min and supernatant was recovered; this represented the cytosolic fraction, while the pellet was suspended in SOL buffer and sonicated to obtain the mitochondrial fraction. To obtain the nuclear fraction, the saved pellet was resuspended in STM + NP-40 (Sigma-Aldrich) buffer, incubated in ice for 30 min, and spun at 500 g for 15 min. The pellet was separated from the supernatant, resuspended in STM buffer, and re-spun at 1000 g for 15 min. The resulting pellet was resuspended in NET buffer and sonicated, producing the nuclear fraction. All buffers contained 3% protease inhibitor cocktail (Sigma-Aldrich, P2714). Protein concentrations of the various fractions were measured using Bradford assay (Bio-Rad Laboratories, Hercules, CA, USA, 5000203). Total protein aliquots (5–15 μg) were resolved in 8–10% Tris-HCL SDS gels. Proteins were electroblotted onto 0.45-μm polyvinyl difluoride (PVDF) membranes (Millipore-Sigma, St. Louis, MO, USA, IPVH00010) with the Mini-Gel Tank and Blot Module Set (Invitrogen, Waltham, MA, USA, NW2000). Membranes were blocked with Intercept blocking buffer (Li-Cor, Lincoln, NE, USA, 927-60001, 927-70001). For the measurement of Nrf2 and BACH1, primary incubations with the corresponding antibody against Nrf2 (1:1000; Proteintech, Rosemont, IL, USA, 16396-1-AP) and BACH1 (1:1000; Proteintech, 14018-1-AP) lasted 16 h. Membranes were then incubated with IRDye 800 CW anti-rabbit (Li-Cor, 926-32213) secondary antibody (Li-Cor, 926-68072) (diluted 1:20,000). Blots were visualized using the Odyssey system (Li-Cor) and quantified using Image Studio Lite ver. 5.2 (Li-Cor). Ponceau stain was used as a loading control following the modified Nakamura’s method [65]. Briefly, membranes were rinsed with distilled water, followed by incubation with the Ponceau staining solution (0.1 [*w*/*v*] Ponceau S powder in 5% [*v*/*v*] acetic acid) for 10 min. Membranes were thoroughly rinsed with distilled water to remove excess stain and to resolve clear bands for imaging.

### 4.5. Biochemical Analyses

Whole cell lysates were prepared by homogenizing 30 mg of tissue in 110 μL of radioimmunoprecipitation assay (RIPA) buffer [66], then incubated in ice for 30 min and centrifuged for 25 min at 10,000 x *g*. The supernatant was recovered representing the whole-cell extract. Total liver 4-HNE was measured from the whole cell extracts (HNE Adduct Competitive ELISA kit, Cell Biolabs, Inc., San Diego, CA, USA, STA-838). The mitochondrial total activity of catalase (CAT; Cayman Chemical, Ann Arbor, MI, USA, 707002), GPx (Cayman Chemical, 703102), and SOD (Cayman Chemical, 706002) were measured in the mitochondrial fraction using commercially available kits as previously described [29,30], following the manufacturer’s instructions. Glucose (Autokit Glucose, Fujifilm Wako Diagnostics, Mountain View, CA, USA, 997-03001) and TAG (Triglyceride Colorimetric Assay Kit, Cayman Chemicals, Ann Arbor, MI, USA, 10010303) were measured from the cytosolic fractions using commercially available kits. All samples were analyzed in duplicate and only values that fell within percent coefficients of variability of less than 10% for all measurements were accepted.

### 4.6. qPCR mRNA Quantification

Total RNA was obtained using TRIzol reagent (Invitrogen, 15596026) from 25 mg of frozen liver, allowing 2 h for the RNA precipitation step. Genomic DNA were degraded using DNase I (Roche, Basel, Switzerland, 04716728001). Complementary DNA were reverse transcribed from genomic DNA-free RNA using the High-Capacity cDNA Reverse Transcription Kit (Applied Biosystems, Waltham, MA, USA, 4368814) using oligo dT. Quantitative PCR (qPCR) reactions were performed in duplicate using the equivalent to 50 ng of RNA per reaction. Specific primers were included in the respective reactions, probing for GCLC, GCLM, GPx1, and GR. Data were normalized using the mRNA levels of beta-2-microglobulin (B2M). The primer sequences used for qPCR analyses are shown in Table 1.

### 4.7. Statistics

Data were tested for normality using the Shapiro–Wilk test [67]. Means (±SD) were considered significantly different at *p* < 0.05 using the Tukey test. Two-way ANOVAs were used when analyzing datasets with all timepoints (T0, T1, T2) and groups, while one-way ANOVAs were used when analyzing datasets without timepoints (for chronic, static effects captured by the results at the T0 measurement period only). Correlations were calculated using the Pearson r coefficient [68] to better assess changes in dynamic responses to glucose over time. Outliers were calculated and removed using the ROUT test [69] and if found, one outlier was replaced using the trimming method as explained by Kwak and Kim [70]. All statistical analyses were performed with GraphPad Prism software (ver. 9.3.1, San Diego, CA, USA).

## Figures and Tables

**Table 1:** Primers sequences used for quantitative PCR.

Primer Name	Sequence	NCBI Reference Sequence
GCLC F GCLC R	AGTGGAGGTAAAAGCGACCC TCACTTGTGGGCAACTGGAA	NM_012815.2
GCLM F GCLM R	GAAAAAGTGTCCGTCCACGC CCACTGCATGGGACATGGTA	NM_017305.2
GR F GR R	CGAGGAAGACGAAATGCGTG CGAAGCCCTGAAGCATCTCA	NM_053906.2
SOD2 F SOD2 R	TTGCTGGAGGCTATCAAGCG CGGCAATCTGTAAGCGACCT	NM_017051.2
Catalase F Catalase R	CACTCAGGTGCGGACATTCT CAGGGTGGACGTCAGTGAAA	NM_012520.2
GPx1 F GPx1 R	GGTGTTCCAGTGCGCAGATA CTTAGGGGTTGCTAGGCTGC	NM_030826.4
IL-1B F IL-1B R	CCTATGTCTTGCCCGTGGAG TCCTGGGGAAGGCATTAGGA	NM_031512.2

## Data Availability

Associated data are available upon reasonable request.
